# Human amniotic membrane: an improvement in the treatment of Medication-related osteonecrosis of the jaw (MRONJ)? A case–control study

**DOI:** 10.1007/s10561-021-09922-y

**Published:** 2021-04-15

**Authors:** Mirko Ragazzo, Matteo Val, Giulia Montagner, Diletta Trojan, Stefano Fusetti, Luca Guarda Nardini

**Affiliations:** 1 Unit of Oral and Maxillofacial Surgery, Ca Foncello Hospital, 31100 Treviso, Italy; 2 Treviso Tissue Bank Foundation, Via dell’Ospedale 3, 31100 Treviso, Italy; 3 Department of Neuroscience, University of Padua, Maxillofacial Surgery Unit, Padova, Italy

**Keywords:** MRONJ, Medication-related osteonecrosis of the jaw, Amniotic membrane, Case–control, Pain reduction

## Abstract

The aim of this article is to report the results obtained by the use of HAM in surgical wound healing and the reduction of relapse in patients affected by Medication-related osteonecrosis of the jaw (MRONJ).The study involved patients with the diagnosis of MRONJ, surgically treated between October 2016 and April 2019, in a case–control setting. Enrolled patients were randomly divided into 2 groups. One group will be treated with resective surgery and with the insertion of HAM patch (Group A), while the second group had been treated exclusively with resective surgery (Group B).The patients underwent MRONJ surgical treatment with the placement of amniotic membrane patches at the wound site. Data regarding the long-term complications/functions were evaluated at 3, 6, 12, and 24 months after surgery. Pain measurements were performed before the intervention (T0), 7(T1) and 30(T2) days after surgery. 49 patients were included in the study. 2 patients of GROUP A after 30 days since they were surgically treated showed persistent bone exposure. 5 patients of group B demonstrated a lack of healing of the surgical wound with the persistence of bone exposed to 30 days after surgery. Statistical analysis ruled out any difference in OUTCOME (relapse) between GROUP A and B (*p* = 0.23). However, the Fisher test highlighted a significant difference between the use of HAM and only surgical treatment in pain at rest (*p* = 0.032). The use of amniotic membrane implement the patient's quality of life and reduce pain perception. has a learning curve that is fast enough to justify its routine use.

## Introduction

Medication-related osteonecrosis of the jaw (MRONJ) is a serious drug-related side-effect, consisting of progressive bone destruction in the maxillofacial region of patients treated with antiresorptive and/or antiangiogenic medications. (Rosella et al. 2016) MRONJ presents major repercussions on the health care system. (Khan et al. 2017) MRONJ is much more common in patients receiving antiresorptive and/or antiangiogenic drugs for cancer-related skeletal events than in patients treated for non-malignant diseases. (Khan et al. 2015) Key factors for the development of MRONJ are the type and dose of antiresorptive and/or antiangiogenic drug, a history of trauma, dental surgery or dental infection. (Khan et al. 2015) Trauma induced by poorly fitting or even adequate removable dentures can lead to chronic irritation of the gingiva and of the underlying alveolar bone and may trigger osteonecrosis. For cancer patients treated with antiresorptive (bisphosphonates (BP)) drugs, the risk of jaw’s osteonecrosis (ONJ) varies between 0 and 6.7%. For patients with osteo metabolic pathology, being treated with BP, the risk of ONJ varies between 0.004 and 0.2%. (Khan et al. 2017; Rosella et al. 2016) Although the disease process of MRONJ remains largely unknown and poorly understood, the dominant hypothesis for the pathogenesis of this condition is that patients receiving antiresorptive and/or antiangiogenic therapies exhibit a diminished bone healing ability, which in turns triggers a cascade of bone necrosis at the site of the traumatic insult in the jaws. (Dodson 2015; Khan et al. 2015) Symptoms and signs of MRONJ range from mild discomfort, erythema, and intraoral bone exposure to pain, swelling, purulence, ulcerations, fistulae, and pathologic fractures. (Sammut et al. 2016) The best treatment practices for the management of patients with MRONJ is largely debated in literature. Different kinds of treatments have been proposed, medical treatments such as antimicrobial mouth rinses, systemic antibiotics, hyperbaric oxygen therapy, pentoxifylline, and teriparatide. (Aghaloo et al. 2015; Fantasia 2015) And also surgical interventions with different degrees of invasiveness: curettage, sequestrectomy, debridement, and surgical resection.(Campisi et al. 2014; Hoff et al. 2008; Ruggiero 2015; Williams and O'Ryan 2015) Due to the absence of guidelines in the literature for the correct management of ONJ, various supports to improve the prognosis and reduce the risk of relapse have been proposed for surgical treatment such as PRF, Buccal fat pad flap, recombinant human BMP-2 and HAM. (Aghaloo et al. 2015; Berrone et al. 2015; El-Rabbany et al. 2018, 2019; Nicolatou-Galitis et al. 2019; Ragazzo et al. 2018) HAM is a tissue obtained from the placenta, which promotes the wound’s healing process due to the high content of growth factors (EGF, FGF, TGF) and tissue metalloprotease inhibitors (TIMP). Furthermore, it has reduced immunogenicity, connected with the reduced presence of HLA-A, B, C or β2 microglobulin antigens. Finally, the anti-inflammatory property of HAM is connected with the capacity to inhibit pro-inflammatory cytokine expressions such as IL-1, IL-2, IL-8, IL-10, and IFN-y. (Paolin et al. 2016) This feature makes it suitable for any type of transplant without the need to start immunosuppressive therapy. HAM appears to be promising in facilitating ONJ post-treatment tissue healing as demonstrated by Ragazzo et al. (2018) The present study evaluates the use of HAM in the management of MRONJ. The aim is to investigate the healing properties and the disease free-survival associated with the use of amniotic membrane. In particular, if these characteristics are influenced by systemic, local or pharmacological factors.

## Materials and methods

### Ethical considerations

This study followed the Declaration of Helsinki on medical protocol and ethics. All patients underwent surgical treatment and follow-up at the Maxillofacial Surgery Unit of the Ca Foncello Hospital in Treviso (Italy). Each patient was informed of the risks associated with surgical treatment and authorized the collection of clinical data and iconographic documentation. Surgical treatment was performed by two oral and maxillofacial surgeons. The study was approved by the local ethics committee with the number “581/CE Marca” and all participants signed an informed consent agreement.

### Study design and patient selection

This is a prospective case–control descriptional study, the control group enrolled patients that underwent a surgical procedure to treat MRONJ without the application of HAM at the site of the wound. The population of this study included all patients who were diagnosed with MRONJ at the Maxillofacial Surgery Unit of the Ca Foncello Hospital in Treviso (Italy) from October 2016 to May 2019 and that didn’t match with the exclusion criteria. Exclusion criteria in the selection of patients for this study were: Patients were not assuming or they had never assumed antiresorptive or antiangiogenic or inhibitor of mTOR drugs; Patients previously underwent radiotherapy of the head-neck region; Patients general conditions didn’t make them possible to undergo surgery.

### Diagnosis of MRONJ

The diagnosis of osteonecrosis was performed for each patient by clinical and anamnestic evaluation. Preoperative orthopantomography, CT and incisional biopsy of the exposed bone, if present, and of the surrounding mucosa were performed. The staging of the lesions was performed according to the SIPMO (Italian Society of Oral Pathology and Medicine)-SICMF(Italian Society for Maxillofacial Surgery) classification of MRONJ (Bedogni et al. 2014, 2012):

#### Stage 1 Focal MRONJ

Clinical signs and symptoms: bone exposure; sudden dental mobility; nonhealing postextraction socket; mucosal fistula; swelling; abscess formation; trismus; gross mandibular deformity and/or hypoesthesia/paraesthesia of the lips.

CT findings: increased bone density limited to the alveolar bone region (trabecular thickening and or focal osteosclerosis), with or without the following signs: markedly thickened and sclerotic lamina dura; persisting alveolar socket; and/orcortical disruption.

- Asymptomatic.
- Symptomatic (pain and purulent discharge).

#### Stage 2 Diffuse MRONJ

Clinical signs and symptoms: same as Stage 1.

CT findings: increased bone density extended to the basal bone (diffuse osteosclerosis), with or without the.

following signs: prominence of the inferior alveolar nerve canal; periosteal reaction; sinusitis; sequestra formation; and/or oro-antral fistula.

- Asymptomatic.
- Symptomatic (pain and purulent discharge).

#### Stage 3 Complicated MRONJ

Same as Stage 2, with one or more of the following: clinical signs and symptoms: extra-oral fistula; displaced mandibular stumps; nasal leakage of fluids.

CT findings: osteosclerosis of adjacent bones (zygoma,hard palate); pathologic mandibular fracture; and/or osteolysis extending to the sinus floor.

### Patient selection

All male and female patients with diagnosis of MRONJ were recruited in the study. Patients were excluded if:

- They are not assuming or they had never assumed antiresoptive or antiangiogenetic or inibitor of mTOR drugs.
- They previously underwent radiotherapy of the head-neck region
- Their general conditions made it impossible to undergo surgery

Enrolled patients were randomly divided into 2 groups. One group will be treated with resective surgery and with the insertion of HAM patch (Group A), while the second group had been treated exclusively with resective surgery (Group B).

### Surgical technique

**Fig. 1:**
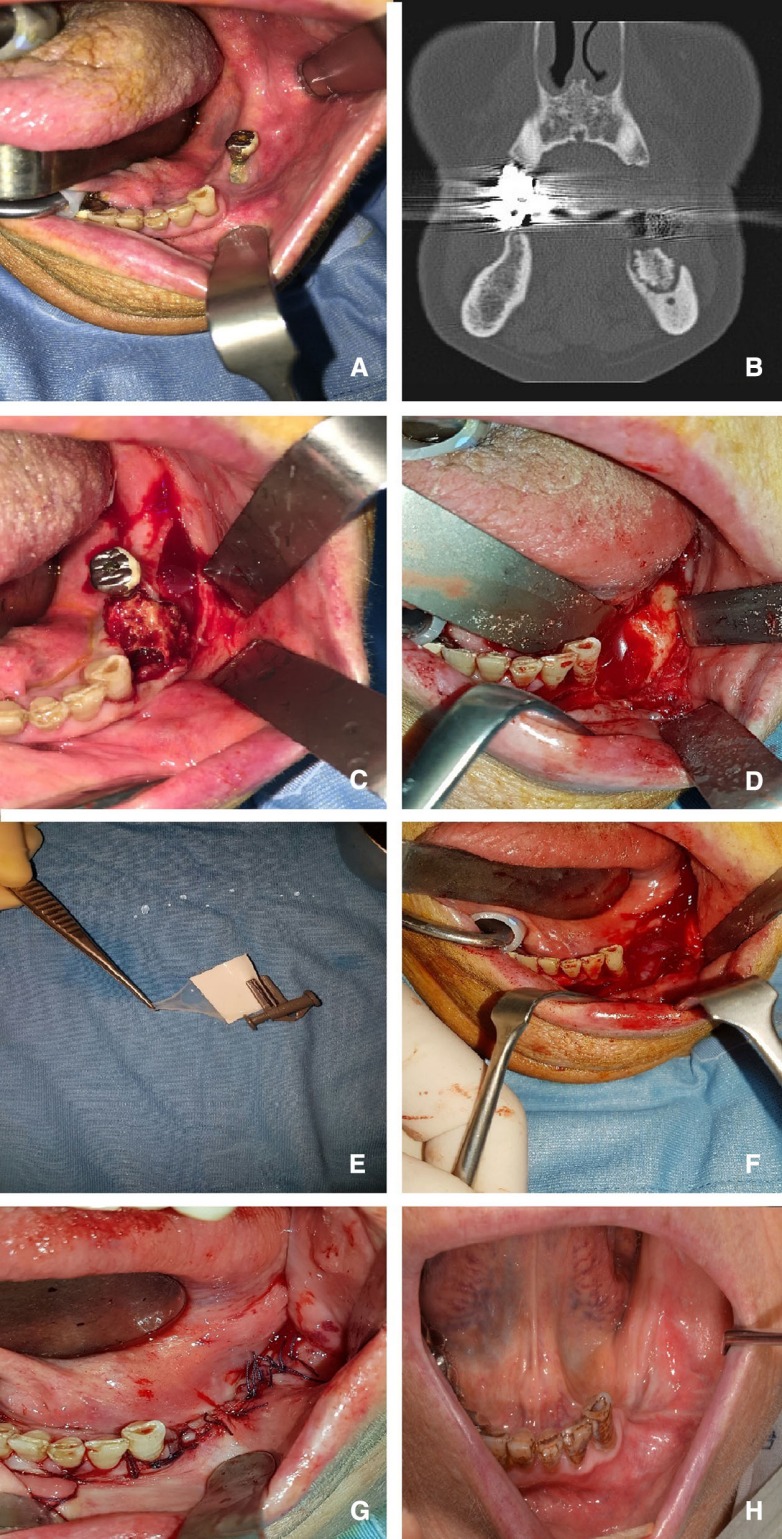
**a** Clinical aspect of MRONJ affected area; **b** Pre-operative TC; **c** Bone sequestrum involving the implant; **d** Curetted Area; **e** HAM; **f** HAM application; **g** suture of mucosal soft tissues and **h** 6 months postoperative follow-up

**Fig. 2:**
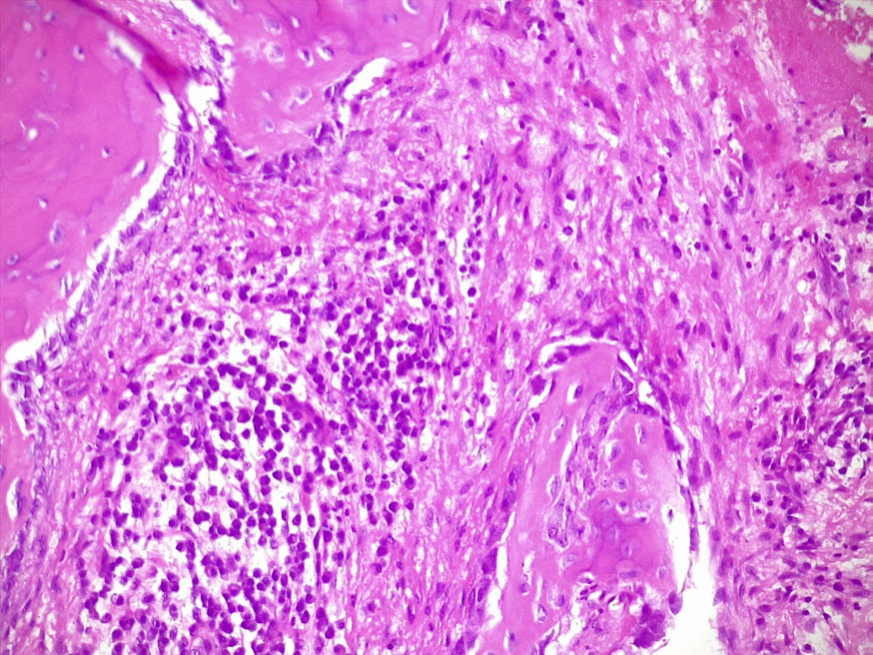
Overview of the oral specimen H&E staining, magnification × 20 of a osteonecrosis with a dense inflammatory infiltrate

All patients were made aware of the benefits and possible complications of the surgical procedure. Prior to surgery, discontinuation of BP drugs was requested at least 1 month before, with the possibility of resuming this treatment once the surgical sites have healed. Patients started antibiotic prophylaxis with amoxicillin and clavulanic acid (875 + 125 mg) and Metronidazole (500 mg), every 8 h, 7 days before surgery and stop it after 7 days post-surgery. Under general anesthesia, after the infiltration of the local anesthetic (mepivacaine), a mucoperiosteal flap was performed. Fistulectomy of the hyperplastic mucosa surrounding the exposed bone was carried out. Furthermore, a debridement of hyperplastic-inflammatory tissue and osteotomy of necrotic bone was performed until fresh bleeding from bone was confirmed. Rotary instruments were used to smoothen out all sharp bony margins. HAM has been placed in the bone defect only in Group B and hermetic suture has been performed to close the surgical wound. Figure 1 (A-G) All resected tissues were sent to the pathologist to obtain the definitive diagnosis. Figure 2 (Ragazzo et al. 2018).

### HAM processing

The placenta is usually sourced from donors undergoing cesarean sections and processed shortly after retrieval. The HAM is carefully detached from the chorion and rinsed with sterile saline solution to remove residual blood. The membrane is flattened on a nitrocellulose membrane filter (Merck Millipore), with its stromal/mesenchymal side facing down, in contact with the filter. Afterward, the HAM is immersed in a cocktail of antibiotics including vancomycin 100 μg/ml (Hospira), meropenem 200 μg/ml (Fresenius Kabi Italia), and gentamicin 200 mg/ml (Fisiopharma) at + 4 °C for 24 h in sterile conditions, validated for human tissues. HAM was cut in 3 × 3 cm^2^ patches and cryopreserved. Microbiological analyses are performed at several stages throughout the process and only HAMs without microbial contamination were considered suitable for implants. (Serafini et al. 2016).

### Study variables

Long-term complications after surgical treatment of ONJ were the predictor variables. The results of the maximum interest for the study were time and modality of healing of the flap and level of pain perceived by the patient during the healing; while inflammation, hematoma, and other complications were considered secondary variables. The third category of variables included age, general condition, pharmacological treatment, site, stage and variables that could be related to the outcome.

### Data collection

Patients’ general state of health, medical history, current medications, clinical and radiographic features of MRONJ were recorded pre-intervention. Data regarding the transient complications were collected 1 week postoperatively. Data regarding the long-term complications were evaluated at 3, 6, 12, and 24 months after surgery. Pain perception was assessed by means of a Visual Analogue Scale (VAS) from 0 to 10, with the extremes being no pain and pain as bad as the patient has ever experienced, at rest, during feeding and phonation. (Hawker et al. 2011) These measurements were performed before the intervention (T0), 7 (T1) and 30 (T2) days after surgery. Ortopantomography was performed at 6 and 12 months postoperatively.

### Statistical analysis

Fisher exact test and the Mann–Whitney U-test were applied, as appropriate. Statistical significance was assumed for a P-value of < 0.05, while values in the range of 0.05 ≤ P < 0.10 were considered as indicating a statistical trend.

## Results

Twentyseven patients were treated surgically with resection of the necrotic bone of the jaw and placement of the HAM patch (Group A). One patient died 1 month after surgery due to a complication related to mammal cancer and was excluded from the study. Twenty patients were female and six were male. Their mean age was 69.48 ± 12.67 ( standard deviation) years (range 36–89 years). The follow-up of these patients ranged from 7 to 42 months. In 45% of patients ONJ event was secondary to a tooth extraction. The percentage of patients who had taken zoledronic acid was 57.69%, 23% of patients were assuming alendronate and the 7.7% ibandronate. While the three remaining patients were taking clodronic acid, and risedronic acid and 1 of them assumed zoledronic and pamidronic acid. The ONJ lesions were located: in the maxilla in seven patients, in the mandible in 16 patients, and in both jaws in 3 patients. Five patients out of 7 had a maxillary sinus involvement. Fifteen patients were suffering for a metastatic malignant disease and were treated with BP drugs administered intravenously; 9 patients with osteoporosis, one with rheumatoid arthritis and another patient suffering for algodystrophy were treated with BPs administered orally (except one patient who was assuming risedronic acid administered intravenously). The average time of administration of the drugs was 23 ± 16 months with a minimum administration period corresponding to 12 months and a maximum of 60 months. At the time of diagnosis, an infectious process involved the MRONJ site in 18 patients while bone exposure was detectable in 22 patients and the pain was referred by 18 patients. From the SIPMO SICMF staging the patients are subdivided: 2 patients have stage 1a disease, 8 patients with stage 1b, 3 patients suffering from stage 2a MRONJ, 11 patients were classified stage 2b and 2 patients were stage 3. In the 7-day postoperative period, the percentage of patients who no longer present pain rises to 92.5% (25 out of 26). 2 patients at 30 days since surgery showed an unhealed surgical wound, so they were successfully retreated. At present (April 2020) 4 patients have died. There are 30 surgical sites in the follow-up and no signs of disease recurrence. The features of the study group were reported in Table 1.

**Table 1:** Group A. Legend: OS = administered orally, IV = intravenous, 1 = positive outcome and 0 = negative outcome, T0 = pre-operative, T1 = 7 days since surgery**,** T2 = 30 days since surgery. Pain was evaluated with VAS scale (1 to 10)

N° of patient	Age	Sex	Diagnosis	BP/Antiangiogenic/antiresorptive	Way of somministration	Site	Staging SICMF-SIPMO	Outcome	Follow-up months
1	65	M	Prostate cancer	Zoledronic Acid	IV	Left mandible	2b	1	34
2	58	F	Lung cancer	Zoledronic Acid	IV	Right mandible	1b	0	12
3	81	F	Osteoporosis	Alendronic Acid	OS	Right maxilla	2b	1	14
4	57	F	Thiroid cancer	Alendronic Acid	OS	Left mandible	1b	0	21
5	78	F	Breast cancer	Zoledronic Acid	IV	Left mandible	2a	1	16
6	60	F	Myeloma	Zoledronic Acid	IV	Left maxilla	2b	1	12
7	62	F	Osteoporosis	Alendronic Acid	OS	Left mandible	1b	1	14
8	58	F	Myeloma	Zoledronic Acid	IV	Left maxilla with sinusitis	3	1	24
9	71	F	Myeloma	Zoledronic Acid	IV	Right maxilla	1b	1	28
10	42	M	Prostate cancer	Zoledronic Acid	IV	Left maxilla with sinusitis	2b	1	18
11	59	F	Breast cancer	Zoledronic Acid	IV	Right mandible	2a	0	11
12	72	F	Osteoporosis	Alendronic Acid	OS	Right maxilla	1b	1	36
13	69	F	Breast cancer	Zoledronic Acid	IV	Left mandible	2b	1	19
14	87	F	Osteoporosis	Clodronicic Acid	OS	Left mandible	2b	1	9
15	64	M	Myeloma	Zoledronic Acid	IV	Left maxilla and mandible	2b	1	26
16	68	F	Myeloma	Zoledronic Acid	IV	Right maxilla	1b	0	32
17	76	F	Breast cancer	Zoledronic Acid	IV	Right mandible	2b	1	16
18	84	F	Breast cancer	Zoledronic Acid	IV	Right mandible	1b	1	22
19	55	F	Osteoporosis	Ibadronic Acid	OS	Left maxilla with sinusitis	2b	1	41
20	68	F	Osteoporosis	Ibadronic Acid	OS	Right maxilla	1b	1	35
21	70	F	Breast cancer	Zoledronic Acid	IV	Right maxilla with sinusitis	3	1	28
22	61	M	Myeloma	Zoledronic Acid	IV	Right and left mandible and maxilla	2b	0	12
23	85	F	Breast cancer	Zoledronic Acid	IV	Right maxilla with sinusitis	2a	1	17

Pain at rest T0	Pain at rest T1	Paint at rest T2	Pain during feeding T0	Pain during feeding T1	Pain during feeding T2	Pain during phonation T0	Pain during phonation T1	Pain during phonation T2
7	0	0	6	0	0	5	0	0
4	6	6	7	0	0	6	0	0
6	0	0	5	0	0	5	0	0
6	6	5	6	0	0	4	0	0
5	0	0	7	0	0	7	0	0
4	0	0	6	0	0	5	0	0
4	4	0	6	0	0	6	0	0
0	0	0	6	0	0	6	0	0
7	0	0	8	0	0	8	0	0
5	0	0	7	0	0	5	0	0
5	5	5	8	0	0	7	0	0
3	0	0	0	0	0	0	0	0
5	0	0	5	0	0	6	5	0
4	3	0	5	0	0	5	0	0
4	0	0	8	0	0	7	0	0
6	6	6	4	0	0	5	0	0
6	0	0	7	7	7	7	7	7
7	5	0	6	6	6	7	6	6
8	0	0	5	0	0	5	0	0
0	0	0	8	0	0	6	0	0
8	0	0	8	0	0	8	0	0
5	5	5	0	0	0	0	0	0
3	0	0	3	0	0	3	0	0

In group B 27 patients were enrolled but 4 died due to complications related to neoplastic disease. The average age of the sample is 67.4 ± 11.04 years. Group B is made up of 4 men and 19 females. 16 patients were assuming zolendronic acid, 4 alendronic acid, 2 ibandronic acid and 1 clodronic acid. 17 suffered from metastatic malignant disease while the remaining 6 suffered from osteoporosis. The characteristics of each patient: the site of manifestation of the ONJ, the stage of ONJ and the trend of pain perception before and after the surgery are shown in Table 2. Five patients of group B demonstrated a lack of healing of the surgical wound with the persistence of bone exposed to 30 days after surgery. All patients were successfully surgically retreated.

**Table 2:** Group B. Legend: OS = administered orally, IV = intravenous, 1 = positive outcome and 0 = negative outcome, T0 = pre-operative, T1 = 7 days since surgery**,** T2 = 30 days since surgery. Pain was evaluated with VAS scale (1–10)

N° of patient	Age	Sex	Diagnosis	BP/antiangiogenic/antiresorptive	Way of somministration	Site	Staging sicmf-sipmo	Outcome	Follow-up months
1	57	F	Algodistrophy	Clodronicic Acid	OS	Left maxilla and right mandibole	1b	1	22
2	51	F	Breast cancer	Zoledronic Acid	IV	Left maxilla with sinusitis	3	1	24
3	79	F	Lung cancer and leucemia	Zoledronic Acid	IV	Right mandible	1b	1	12
4	63	F	Osteoporosis	Alendronic Acid	OS	Mandible from 3.7 to 4.7	2b	1	14
5	89	F	Osteoporosis	Alendronic Acid	OS	Mandible symphysis	2b	1	12
6	53	M	Myeloma	Zoledronic Acid	IV	Rght maxilla with sinusitis	2b	1	20
7	83	F	Osteoporosis	Alendronic Acid	OS	Left mandible with cutaneous fistula	3	1	10
8	65	F	Osteoporosis	Alendronic Acid	OS	Left mandible	1b	1	20
9	75	M	Protate cancer	Zoledronic Acid	IV	Left mandible	1a	1	36
10	36	M	Myeloma	Zoledronic Acid	IV	Left maxilla and mandible	2b	1	21
11	57	F	Lung cancer	Zoledronic Acid	IV	Right maxilla	2b	1	7
12	86	F	Osteoporosis	Ibadronico Acid	OS	Right mandible	1b	1	40
13	73	F	Breast cancer	Zoledronic Acid	IV	Left mandible	1b	1	22
14	84	M	Osteoporosis	Alendronic Acid	OS	Left mandible	2b	1	7
15	82	F	Myeloma	Zoledronic Acid	IV	Left mandible	2b	1	37
16	56	F	Myeloma	Zoledronic Acid	IV	Right and left mandible and left maxilla	1a	1	36
17	60	F	Breast cancer	Ac. Zoledronico + Pamidronic Acid	IV	Right and left mandible and maxilla	1b	0	13
18	72	M	Thiroid cancer	Zoledronic Acid	IV	Right mandible	2b	0	20
19	76	F	Reumatoid artritis	Zoledronic Acid	IV	Right maxilla with sinusitis	2a	1	22
20	77	F	Osteoporosis	Ibadronico Acid	OS	Right mandible	2b	1	24
21	79	F	Osteoporosis	Alendronic Acid	OS	Right maxilla with sinusitis	2b	1	21
22	66	F	Myeloma	Zoledronic Acid	IV	Right maxilla with sinusitis	2a	1	28
23	71	F	Breast cancer	Zoledronic Acid	IV	Right mandible	1b	1	14
24	59	F	Myeloma	Zoledronic Acid	IV	Left mandible	1b	1	25
25	77	F	Osteoporosis	Risendronic Acid	IV	Left maxilla	2a	1	24
26	72	M	Myeloma	Zoledronic Acid	IV	Left mandible	2b	1	42

Pain at rest t0	Pain at rest t1	Paint At Rest T2	Pain during feeding T0	Pain during feeding T1	Pain during feeding T2	Pain during phonation T0	Pain during phonation T1	Pain during phonation T2
5	0	0	6	0	0	5	0	0
6	0	0	7	0	0	6	0	0
4	0	0	5	0	0	5	0	0
5	0	0	6	0	0	4	0	0
6	0	0	7	0	0	7	0	0
5	0	0	6	0	0	5	0	0
5	0	0	6	0	0	6	0	0
5	0	0	6	0	0	6	0	0
1	0	0	4	0	0	3	0	0
6	0	0	7	0	0	5	0	0
7	0	0	8	0	0	7	0	0
0	0	0	0	0	0	0	0	0
4	0	0	5	0	0	6	5	0
4	0	0	5	0	0	5	0	0
6	0	0	8	0	0	7	0	0
2	0	0	4	0	0	2	0	0
5	7	7	7	7	7	7	7	7
5	5	5	6	6	6	7	6	6
3	0	0	4	0	0	2	0	0
6	0	0	8	0	0	6	0	0
8	0	0	8	0	0	8	0	0
0	0	0	0	0	0	0	0	0
3	0	0	3	0	0	3	0	0
5	0	0	7	0	0	6	0	0
1	0	0	4	0	0	5	0	0
4	0	0	4	0	0	4	0	0

**Fig. 3:**
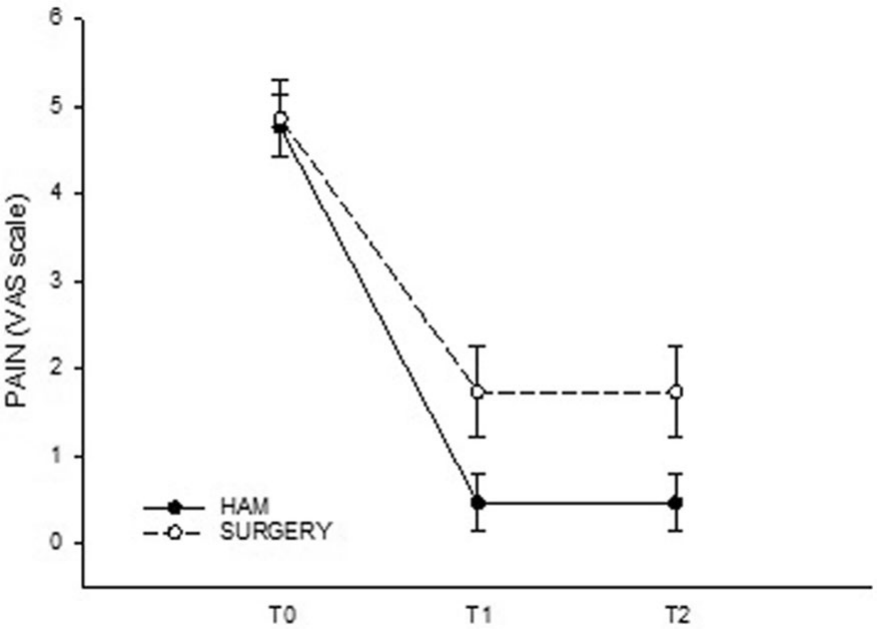
Pain at rest between Group A and B

In Table 3 and 4 are showed the descriptive statistic datas of the two groups of patients. It was not possible to highlight a statistical correlation between the outcome of the surgery and the type of treatments (*p* = 0.23 Fisher test), probably, due to the small sample of patients. If we exclude the 2 "non-responders" patients of the HAM group in the evaluation of the reduction in pain, the use of the amniotic membrane was statistically significant in reducing the pain perception at rest in the post-operative period (*p* = 0.010; Two Way Repeated Measures ANOVA test). If we consider the absence of pain (Y) and the presence of pain (N) in pain at rest T1 as outcome, the Fisher test highlights a statistically significant correlation between the use of HAM and the reduction of pain (*p* = 0.032). (Fig. 3) Pain at rest T1 if compared in group A and B with a Mann–Whitney Rank Sum Test showed that pain reduction in HAM is still significant (*p* = 0.028).

**Table 3:** Statistical descriptive datas of GROUP A

Column	Size	Missing	Mean	Std Dev	Std. error	C.I. of mean
HAM, VAS T0	26	0	4.769	1.796	0.352	0.725
HAM, VAS T1	26	0	0.462	1.655	0.325	0.668
HAM, VAS T2	26	0	0.462	1.655	0.325	0.668

**Table 4:** Statistical descriptive datas of GROUP B

Column	Size	Missing	Mean	Std dev	Std. error	C.I. of mean
Surgery, VAS T0	23	0	4.870	2.096	0.437	0.906
Surgery, VAS T0 T1	23	0	1.739	2.508	0.523	1.085
Surgery, VAS T2	23	0	1.174	2.289	0.477	0.990

## Discussion

HAM has numerous properties, first of all, it is poorly immunogenic (Umezawa et al. 2019) and consequently does not cause transplant rejection in the recipient. Then HAM has anti-inflammatory, antiangiogenic properties and regulates tissue scarring, promoting the healing process thanks to growth factors (EGF, TGF, PDGF, FGF). (Hashim et al. 2016; Umezawa et al. 2019) The expression of these factors, such as the TGF, is preserved even during cryopreservation of the amniotic membrane at -80 °C. (Koizumi et al. 2000) HAM inhibits pro-inflammatory cytokine expression such as IL-1, IL-2, IL-8, IL-10, and IFN-y; (Hashim et al. 2016; Umezawa et al. 2019) while its antiangiogenic action is achieved through the production of endostatin and tissue inhibitors of metalloproteinases (TIMP-1, 2, 3 and 4). (Hao et al. 2000) The poor immunogenicity was initially thought to be related to the absence of antigens of the major histocompatibility complex HLA-A, B or DR. (Adinolfi et al. 1982) Subsequent studies have shown that antigen expression HLA class Ia complex (HLA-A, B C, DR) and Ib (HLA-E, G) is very limited on the epithelial and mesenchymal faces of the membrane. (Houlihan et al. 1995) The antimicrobial impact of AM and amniotic fluid is attributable to the presence of bactricidin, beta-lysin, lysozyme, transferrin and 7-S immunoglobulins in the amniotic fluid. (Galask and Snyder 1970) HAM seems to be able to reduce the pain experienced by patients. The adherence of the amnion to the surgical wound and the coverage of nerve endings is the basis of this phenomenon.(Kesting et al. 2014) This feature makes HAM suitable in oral and maxillofacial surgery, where it has been used since 1969 for the management of mucous defects after resection of malignant or precancerous lesions, due to the closure of oro-antral communication, for guided bone regeneration, post-traumatic orbital surgery and temporomandibular joint surgery.(Guarda-Nardini et al. 2019; Kesting et al. 2014) The present work carries on what had already been proposed in a case report (Ragazzo et al. 2018) of the Authors of this paper. Because the main pharmacologic effect of BP is inhibition of osteoclasts and of bone vascularization, HAM was expected to stimulate both soft tissue healing and bone remodeling, thus contributing to the successful treatment of MRONJ.

Guidelines do not recommend surgery as the first approach in the treatment of early-stage MRONJ, advising to continue with conservative therapy indefinitely or until the progression of the disease. Surgical treatment of ONJ with HAM application increases long-term success compared to medical treatment. (Campisi et al. 2014; El-Rabbany et al. 2018, 2019; Lopes et al. 2015) There are many studies that demonstrate the success of surgical management of these lesions compared to only pharmacological treatment.(Mucke et al. 2011; Wilde et al. 2011) Several authors have also highlighted better results with larger resections than bone debridement.(Bedogni et al. 2011; Ngamphaiboon et al. 2011; Reich et al. 2015) Reich et al. had shown an efficacy of surgery in the treatment of ONJ equal to 83.6% (average follow-up 23.5 months) (Reich et al. 2015); while Bedogni et al. in a group of 30 patients pharmacological treated with BF, who underwent surgical treatment due to MRONJ highlighted a recurrence rate of 3.1% and 9.4% at 3 and 6 months, respectively.(Bedogni et al. 2011) Group B (control) of our study demonstrates similar characteristics with other study group in the literature (Bedogni et al. 2011) treated with surgery alone. The use of the amniotic membrane, form the descriptive analysis of the data, seems to reduce the pain perception in the post-operative period and have a lower risk of a dehisced wound in the post-operative period. Our study has shown that with the use of HAM the success rate of the surgical treatment of 30 sites affected by ONJ is equal to 90% (only 3 sites showed a relapse). The outcomes of our work are similar to those obtained in the treatment of MRONJ with the use of PRF.(Norholt and Hartlev 2016; Park et al. 2017) To our knowledge no study has evaluated the trend of postoperative pain in patients treated for ONJ. In our cohort HAM seemed to have a good ability to reduce pain since the immediate post-surgery. The use of HAM in the surgical management of MRONJ can become mandatory to improve patient comfort.

## Conclusion

In conclusion, our study shows that surgical resection and the use of HAM might be effective in the treatment of MRONJ, in particular, it seems to stimulate soft tissue healing and reducing pain perception in the post-operative period. In spite of these results, it is worth further investigating the role of HAM in the management of ONJ in a larger number of patients and if it could be useful in daily practice.

## Data Availability

The datasets generated during and/or analysed during the current study are available from the corresponding author on reasonable request.
